# Efficient Generation of iPS Cells from Skeletal Muscle Stem Cells

**DOI:** 10.1371/journal.pone.0026406

**Published:** 2011-10-18

**Authors:** Kah Yong Tan, Sarah Eminli, Simone Hettmer, Konrad Hochedlinger, Amy J. Wagers

**Affiliations:** 1 Section on Islet Cell and Regenerative Biology, Joslin Diabetes Center, Boston, Massachusetts, United Stated of America; 2 Department of Stem Cell and Regenerative Biology, Harvard University, Cambridge, Massachusetts, United Stated of America; 3 Harvard Stem Cell Institute, Cambridge, Massachusetts, United Stated of America; 4 Department of Molecular and Cellular Biology, Harvard University, Cambridge, Massachusetts, United Stated of America; 5 Massachusetts General Hospital Cancer Center and Center for Regenerative Medicine, Boston, Massachusetts, United Stated of America; 6 Department of Biology, Chemistry and Pharmacy, Free University of Berlin, Berlin, Germany; 7 Department of Pediatric Oncology, Dana Farber Cancer Institute and Division of Pediatric Hematology/Oncology, Children's Hospital, Boston, Massachusetts, United Stated of America; 8 Howard Hughes Medical Institute, Chevy Chase, Maryland, United States of America; Leiden University Medical Center, Netherlands

## Abstract

Reprogramming of somatic cells into inducible pluripotent stem cells generally occurs at low efficiency, although what limits reprogramming of particular cell types is poorly understood. Recent data suggest that the differentiation status of the cell targeted for reprogramming may influence its susceptibility to reprogramming as well as the differentiation potential of the induced pluripotent stem (iPS) cells that are derived from it. To assess directly the influence of lineage commitment on iPS cell derivation and differentiation, we evaluated reprogramming in adult stem cell and mature cell populations residing in skeletal muscle. Our data using clonal assays and a second-generation inducible reprogramming system indicate that stem cells found in mouse muscle, including resident satellite cells and mesenchymal progenitors, reprogram with significantly greater efficiency than their more differentiated daughters (myoblasts and fibroblasts). However, in contrast to previous reports, we find no evidence of biased differentiation potential among iPS cells derived from myogenically committed cells. These data support the notion that adult stem cells reprogram more efficiently than terminally differentiated cells, and argue against the suggestion that “epigenetic memory” significantly influences the differentiation potential of iPS cells derived from distinct somatic cell lineages in skeletal muscle.

## Introduction

Skeletal muscle is a complex tissue composed primarily of multinucleated fibers, but also containing at least two distinct stem cell populations (muscle-forming satellite cells and non-myogenic mesenchymal precursors). Self-renewing muscle satellite cells can be isolated by fluorescence activated cell sorting (FACS) on the basis of their expression of a unique constellation of cell surface markers [1]. CD45− Mac1− Sca1− β1-integrin+ CXCR4+ satellite cells, (hereafter referred to as skeletal muscle precursors, or “SMPs”) express the canonical satellite cell transcription factor PAX7, and exhibit muscle-specific stem cell activity in both *in vitro* clonogenic assays and *in vivo* transplant settings [2], [3], [4]. The differentiated daughters of SMPs (myoblasts) can likewise be found in association with skeletal myofibers in adult muscle and isolated by surface marker staining and cell sorting [4], but these cells express increased levels of muscle differentiation markers and are unable to engraft mature myofibers or reconstitute the satellite cell compartment upon intramuscular transplant. Finally, adult muscle is also home to a developmentally distinct population of bipotent, mesenchymal progenitors, which are marked by surface expression of Sca-1 [2], [3] and undergo both fibrogenic and adipogenic differentiation *in vitro* and *in vivo* [2],[3],[5],[6].

Recent studies indicate that while reprogramming of somatic cells into induced pluripotent stem (iPS) cells generally occurs at low efficiency, immature blood stem and progenitor cells reprogram very efficiently (up to 28% of input cells; [7]), compared to their terminally differentiated daughters. Intriguingly, iPS cells derived from hematopoietic lineages were reported to exhibit biased differentiation to form blood cells in hematopoietic colony-forming assays [8], and a recent study reported similar lineage-biased differentiation among iPS cells reprogrammed from blood vessel-associated mesoangioblasts [5]. Thus, to test whether adult stem cells in other mesodermal tissues likewise exhibit superior reprogramming efficiency and retain an epigenetic memory that biases their differentiation potential, we examined reprogramming and differentiation capacity among stem and progenitor cells of the myogenic [2], [4] or fibrogenic/adipogenic [3], [5] lineages found in adult mouse skeletal muscle. Our data indicate that, as in the hematopoietic system, myogenic and fibrogenic/adipogenic stem cells show enhanced reprogramming efficiency in comparison to their differentiated daughters. However, in contrast to prior reports [5], [8], [9], we find no evidence for biased differentiation among SMP-derived iPS cells. These data support the existence of cell-intrinsic barriers to efficient reprogramming, which are raised during the process of tissue-specific differentiation, and argue against suggestions that lineage-specific epigenetic marks left behind following reprogramming significantly restrict the developmental potential of somatically derived iPS cells.

## Results

### Isolation of myofiber-associated cells from a reprogrammable mouse for reprogramming

To investigate the reprogramming efficiency of muscle-resident stem and progenitor populations, we expressed the four canonical reprogramming factors (Oct4, Sox2, Klf4, c-Myc) [10] using a “secondary reprogramming system” [11], [12], [13]. In this system, primary iPS cells were generated first by infection of neonatal tail-tip fibroblasts, carrying the ROSA-rtTA transactivator, with lentiviruses expressing Oct4, Sox2, Klf4 and cMyc, each under the control of a doxycycline (dox)-inducible promoter (Figure S1) [14]. The resulting iPS cells were injected into mouse blastocysts, where they produced fetal liver cells that were then differentiated *in vitro* into CD8^+^ cells, and then re-induced with dox to produce (secondary) iPS cells that were used to generate “reprogrammable mice” [13], [14]. Cells harvested from these mice could be converted into iPS cells upon exposure to dox (which activates re-expression of the integrated reprogramming factors in the transgenic cells of these animals). Importantly, the integrated reprogramming factors in these chimeric mice are maintained in an identical genomic configuration in all iPS cell-derived somatic cells. Therefore this system allows direct comparison of the reprogramming efficiencies of distinct cell populations and lineages harvested from these mice, without potential complications arising from differences in lentiviral transduction efficiency or variation in integration sites.

**Figure 1:**
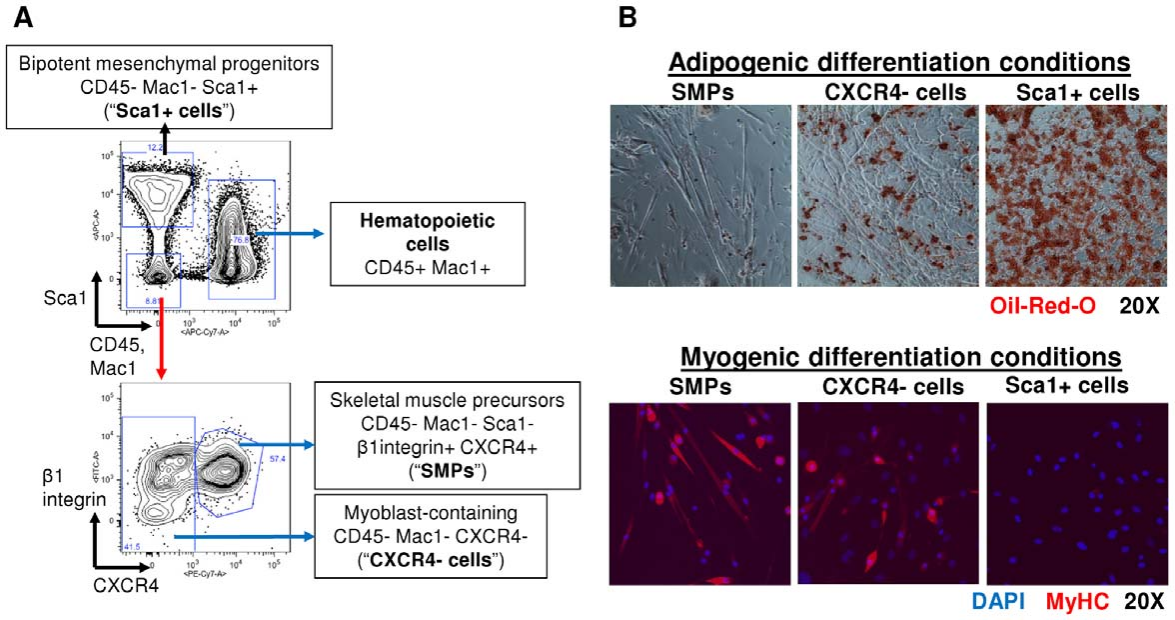
Isolation and differentiation potential of myofiber-associated skeletal muscle precursors (SMPs) and Sca-1+ mesenchymal progenitors. (**A**) Myofiber-associated cells were isolated from mouse hind limb muscles and triceps. SMPs were isolated by FACS as CD45− Mac1− Sca1− β1-integrin+ CXCR4+, while bipotent, adipogenic/fibrogenic cells were identified and isolated as CD45− Mac1− Sca-1+ (abbreviated Sca1+; [3], [6]). All cell populations were double-sorted, yielding highly purified cell populations that subsequently were seeded onto irradiated MEFs in the presence of doxycyline (see Figure 2). (**B**) SMPs but not Sca1+ cells can differentiate into myosin heavy chain+ (MyHC+) cells, indicating their myogenic differentiation potential (bottom panel). Conversely, Sca1+ cells but not SMPs can form adipogenic cells, as indicated by Oil-Red-O staining (top panel). CXCR4− cells are a mixture of myoblasts (bottom panel) and fibroblasts, some of which can adopt an adipocytic fate under adipogenic culture conditions (top panel).

SMPs were isolated by two-step enzymatic digestion of skeletal muscle, followed by fluorescence activated cell sorting (FACS) for the cell surface marker profile: CD45^−^ Mac-1^−^ Sca-1^−^ β1-integrin^+^ CXCR4^+^ (CSM4B) (Figure 1A) [2], [4]. Consistent with previous publications [2], [4], [6], [15] , isolated SMPs showed potent myogenic potential and were never observed to form fibroblasts or adipocytes, even under adipogenic culture conditions (Figure 1B). In addition to SMPs, we also isolated from skeletal muscle a distinct population of non-myogenic CD45^−^ Mac-1^−^ Sca-1^+^ mesenchymal progenitor cells, referred to here as “Sca1^+^ cells”, which both self-renew and possess bipotent fibrogenic and adipogenic differentiation potential [2],[3],[5],[6] (Figure 1). Finally, to assess the effects of differentiation on reprogramming efficiency, we isolated a third population of CD45^−^ Mac-1^−^ Sca-1^−^ CXCR4^−^ cells, hereafter referred to as “CXCR4^−^ cells”, which is composed of differentiated myoblasts and fibroblasts that lack self-renewal activity (Figure 1 and [4]). This approach allowed us to directly compare the reprogramming potential of three distinct cell populations residing in an identical microenvironmental “niche”, including both lineally distinct self-renewing tissue stem cells (SMPs and Sca1^+^ cells) and the more differentiated, non-self-renewing progeny of such cells (CXCR4^−^ cells).

### SMPs and bipotent fibrogenic/adipogenic progenitors reprogram efficiently

**Figure 2:**
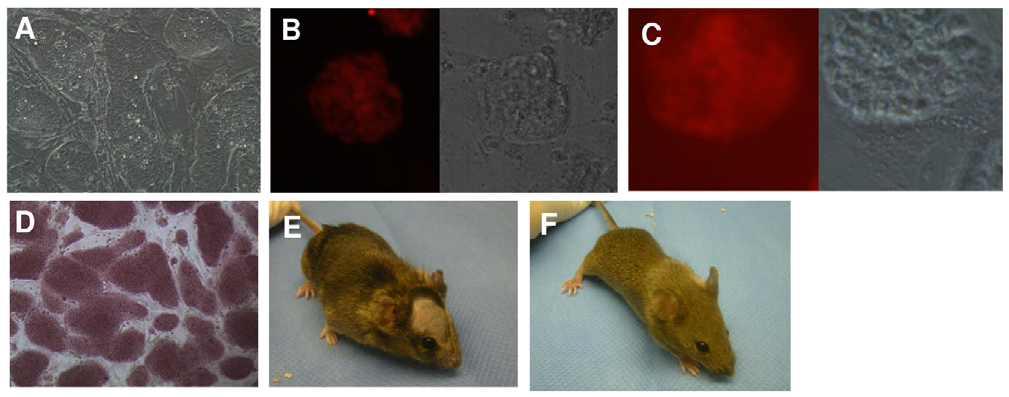
SMPs and Sca-1+ mesenchymal progenitors reprogram into pluripotent iPS cells with high efficiency. (**A–F**) iPS cells generated from SMPs (“SMP iPS” cells) show embryonic stem cell-like morphology (**A**), and stain for the pluripotency markers Oct 4 (**B**) and SSEA-1 (**C**). Brightfield and corresponding fluorescence images (red) are shown for B and C. SMP iPS cells also show alkaline phosphatase activity (**D**). When SMP iPS cells were injected into C57Bl/6 blastocysts, the resulting chimeras had agouti coat color (**E**), indicating contribution of the SMP iPS cells to chimeric tissues. Offspring from matings between SMP iPS cell chimeric mice and C57Bl/6 mice have brown coat color (**F**), demonstrating the capacity of SMP iPS cells to provide germline transmission.

To quantify reprogramming efficiencies, iPS colonies arising from each dox-induced cell population were identified by morphological criteria and counted at day 21 after dox induction. We observed a significantly greater number of iPS colonies emerging from seeded SMPs, compared to more differentiated CXCR4^−^ cells. In particular, while seeded SMPs formed iPS colonies with an average uncorrected reprogramming efficiency of 1.8% (n = 2 experiments, range = 0.7–2.8%) and Sca1+ cells formed iPS colonies with an average uncorrected reprogramming efficiency of 0.04% (n = 2 experiments, range = 0.01–0.07%), we obtained no iPS colonies from CXCR4^−^ cells (Figure S2). Importantly, the iPS cell lines generated from both SMPs and Sca-1^+^ cells were demonstrated to be pluripotent by *in vitro* staining for the pluripotency markers Oct-4, SSEA-1 and alkaline phosphatase (Figure 2A–D). Moreover, blastocyst injection of two “SMP-iPS” lines each produced mice showing a high degree of SMP-iPS cell chimerism, including germline contribution and transmission (Figure 2E,F, and data not shown).

The data presented above seem to indicate a significantly increased rate of successful iPS cell derivation from tissue-resident stem/progenitor cells (both SMPs and Sca1+ cells) in comparison to their differentiated daughters (CXCR4− cells) residing in the same anatomical location; however, given that the chimeric mice from which SMPs and CXCR4− cells were isolated for these initial experiments contained both transgenic (iPS-derived, containing the dox-inducible reprogramming factors) and non-transgenic (host blastocyst-derived) cells, the uncorrected reprogramming efficiencies reported above likely underestimate the true efficiency of target cell reprogramming. To more accurately determine the reprogramming efficiency of these cells, in a subsequent series of experiments, we utilized reprogrammable mice carrying the four doxycycline-inducible transgenic reprogramming factors, as before, but additionally labeled the pluripotent cells that gave rise to these chimeric mice with a lentivirus constitutively-expressing tdTomato [14]. This strategy allowed us to prospectively isolate only the transgenic “reprogrammable” cells, thereby enabling more accurate determination of reprogramming efficiency (Figure S3). Using this improved system, we found that the average efficiency of derivation of iPS colonies from bulk-sorted tdTomato^+^ SMPs was ∼10% after 3 weeks (n = 2 independent experiments), whereas again no iPS colonies arose from CXCR4^−^ cells (n = 3 independent experiments), suggesting that their reprogramming efficiency is too low to be detected under our experimental conditions (Table 1).

**Table 1:** TdTomato+ skeletal muscle precursors (SMPs), myoblasts (CXCR4− cells) and bipotent mesenchymal progenitors (Sca1+ cells) were sorted onto irradiated MEFs.

Cell type	Efficiency of reprogramming % (No of iPSC colonies/No of cells seeded)			Average efficiency of reprogramming +/− standard error of mean
	Expt 1	Expt 2	Expt 3	
Skeletal muscle progenitors (Sca1− CD45− Mac1− β1-integrin+ CXCR4+; “SMPs” in text)	11% (46/430)	10% (30/300)	ND	10.3+/−0.3%
Mesenchymal progenitors (Sca1+ CD45− Mac1−; “Sca1+ cells” in text)	ND	9% (28/300)	10% (48/500)	9.5+/−0.1%
Myoblast-containing population (Sca1− CD45− Mac1− CXCR4−; “CXCR4− cells” in text)	0% (0/1352)	0% (0/300)	0% (0/500)	0+/−0%

Dox was added to induce transgenic expression of Oct4, Sox2, Klf4 and c-Myc. After three weeks, the number of iPS colonies with embryonic stem cell-like morphology was counted. Data are presented as a percentage of input cells and number of iPS colonies per number of cells seeded. ND: not determined.

### Single-cell cloning confirms higher SMP and Sca1+ cell reprogramming efficiency

**Figure 3:**
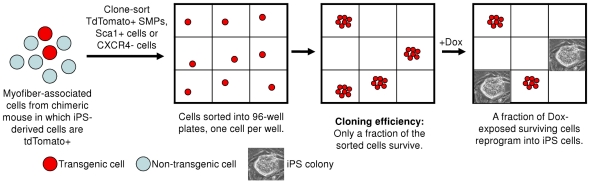
Clone-sorted myofiber-associated stem cells reprogram at high efficiency. (**A**) Experimental strategy for clone-sorting and reprogramming sorted cells. Myofiber-associated cells from transgenic mice carrying dox-inducible transgenes (Oct4, Sox2, c-Myc, Klf4) and labeled with constitutively-expressed Tdtomato [22], were double-sorted for purity, and seeded into 96-well plates, at one cell per well, on irradiated MEFs. After three weeks in dox-containing media, emergent colonies were trypsinized and passaged in the absence of dox, and iPS lines were established which showed embryonic stem cell-like morphology, stained for the pluripotent marker SSEA-1, and showed alkaline phosphatase activity (data not shown).

To exclude the possibility that the study described above might overestimate the reprogramming efficiency of target cell populations after bulk-sorting, by counting “satellite colonies” emerging from primary iPS colonies, we next performed clonal assays in which TdTomato^+^ SMPs, Sca1^+^ cells, or CXCR4^−^ cells were sorted at one cell per well in a 96-well format (Figure 3). To ensure accurate deposition of a single cell in each well, cells were sorted directly into wells that already contained irradiated MEFs, and reprogrammed by addition of doxycycline-supplemented medium. After 2–3 weeks, colonies with iPS morphology emerged in several wells, and with subsequent passaging, these colonies developed into dox-independent iPS lines (as verified by *in vitro* staining of pluripotency markers, data not shown). In these clonal assays, SMPs gave rise to iPS lines at a frequency of 3–10% (number of iPS colonies derived divided by the number of wells seeded with SMPs), while again no iPS colonies were detected in wells seeded with differentiated CXCR4^−^ cells (Table 2, column 3).

**Table 2:** The relative efficiencies of reprogramming for each population are shown, before and after normalization for cloning efficiency.

Cell type		No. iPS colonies/no. input cells (Reprogramming efficiency %)	Cloning efficiency	Reprogramming efficiency (Normalized for cloning efficiency)	Mean efficiency +/− S.E.M.
**SMPs**	Expt 1	18/300 (6.0%)	25%	24%	23+/−3%
	Expt 2	4/120 (3.3%)	20%	17%	
	Expt 3	5/120 (4.2%)	15%	28%	
	Expt 4	6/60 (10.0%)	ND	-	n/a
**Sca1+**	Expt 1	4/120 (3.3%)	15%	22%	29+/−7%
	Expt 2	5/120 (4.2%)	12%	36%	
	Expt 3	2/60 (3.3%)	ND	-	n/a
	Expt 4	4/60 (6.6%)	ND	-	n/a
**CXCR4−**	Expt 1	0/120 (0.0%)	12%	0%	0%
	Expt 2	0/120 (0.0%)			

Cloning efficiency of sorted cells was determined by plating each of the indicated cell types into 96-well plates, one cell per well, and counting the number of wells containing live cells after 7 days. ND: not determined.

However, previous work informed us that single-cell sorting and plating of SMPs in 96-well plates does not occur with absolute efficiency [2], [4]. Hence, we also determined the clonal plating efficiency of SMPs by seeding single-cells into 96-well plates without inducing reprogramming, and counting the number of wells containing live cells after a week. Plating efficiencies of SMPs varied from 15–25% (Table 2, column 4), and so, using similar plating efficiencies for cells sorted under induced reprogramming conditions, we calculate that the actual efficiency of iPS derivation from SMPs averages 23% (±3%), and can reach up to 28% (Table 2, columns 5 and 6). We obtained similar reprogramming efficiencies with Sca1^+^ cells (Table 2). Thus, in light of the fact that the differentiated progeny of SMPs and Sca-1^+^ cells – CXCR4^−^ myoblasts and fibroblasts – reprogram at very low to undetectable efficiencies (Figures 2 and 3; note also that, using this identical system, Eminli et al. (2009) reported a reprogramming efficiency of differentiated fibroblasts from tail tip of only 0.74%), these data support the notion that adult stem and progenitor cells may be more amenable, in general, to reprogramming into iPS cells than their terminally differentiated counterparts.

**Figure 4:**
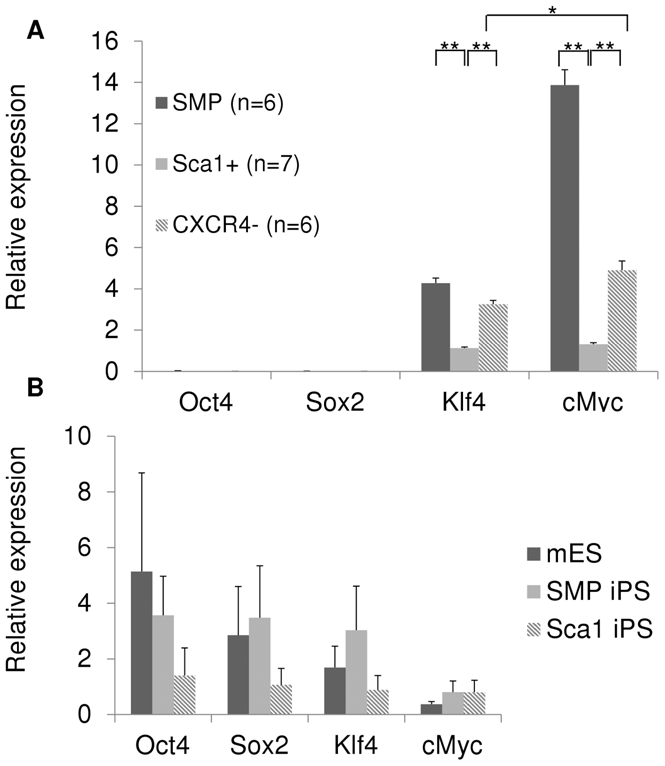
Increased efficiency of reprogramming of SMPs and Sca1+ cells is independent of initial levels of endogenous expression of reprogramming factors. Quantitative real-time PCR using primers that detect endogenously expressed Oct4, Sox2, Klf4, and cMyc mRNAs was performed on freshly sorted SMPs, Sca1+ cells and CXCR4− cells (**A**) and on reprogrammed SMP and Sca1+ iPS cells and mES cells (three different lines tested for each cell type, **B**). None of the freshly-isolated myofiber-associated cell populations expresses Oct4 or Sox2 at appreciable levels compared to pluripotent cells. SMPs and CXCR4− cells express Klf4 and c-Myc at significantly higher levels than in Sca1+ cells. Data are normalized for beta2-microglobulin expression and presented as mean +/− S.E.M. for the indicated number of replicates. *p<0.05; **p<0.001.

Significantly, the higher efficiency of reprogramming of SMPs and Sca1+ cells was not attributable to higher endogenous expression of reprogramming factors (Oct4, Sox2, Klf4 and c-Myc). Quantitative real-time PCR, performed on freshly isolated cell populations, revealed that none of the myofiber-associated cell populations studied here expresses appreciable levels of Oct4 or Sox2, as compared to pluripotent cells (Figure 4). In addition, SMPs and CXCR4− cells, which show discrepant reprogramming efficiency (Figures 2 and 3), expressed Klf4 and c-Myc at levels significantly greater than Sca1+ cells, which show an efficiency of reprogramming equivalent to SMPs, and much greater than differentiated CXCR4− cells (Figures 2, 3, and 4). Likewise, although previous reports have suggested that proliferation rate may influence the reprogramming efficiency of somatic cells [16], [17], we found no significant differences in the rate of proliferation of SMPs, Sca1+ cells and CXCR4− cells, based on direct cell counting and on incorporation of bromodeoxyuridine in cultured cells (data not shown). These data are consistent with our previous studies [7], in which varying proliferation rates of hematopoietic precursor cells did not affect reprogramming efficiency. Thus, these data indicate that reprogramming efficiency does not correlate with endogenous expression levels of reprogramming factors, suggesting that other attributes, unique to adult stem cell populations, are responsible for the enhanced reprogramming efficiency of these cells.

### Teratomas formed from SMP iPS cells do not display biased differentiation towards somatic cell lineage of origin

Recent reports have suggested that early passage iPS cells retain an “epigenetic” memory of their somatic cell of origin, which influences their subsequent differentiation potential [8], [18]. Therefore, we sought to investigate if differences in cell-of-origin might translate into differences in muscle lineage differentiation capacity for iPS cells generated from myogenic SMPs or from non-myogenic Sca1+ cells. iPS cells generated from these two different cells-of-origin were compared to conventionally derived mES cells following subcutaneous injection into immunocompromised NOD/SCID mice. These experiments used only early passage iPS cells (3–5 passages after emergence from reprogramming conditions), as iPS cells have been reported to lose epigenetic markers of their somatic cell-of-origin after 5–6 passages [8].

**Figure 5:**
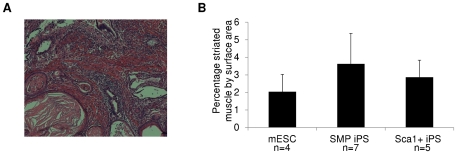
Teratomas from SMP iPS and Sca1+ iPS cells do not show biased differentiation into skeletal muscle by histology. (**A**) Representative image of an H&E stained section of a teratoma formed from SMP iPS cells (A). The presence of skeletal muscle in teratomas from mES, SMP iPS or Sca1+ iPS was assessed by histology, and is shown as the percent area of the tumor occupied by muscle (B). No significant differences were detected in the fraction of striated muscle present in teratomas derived from mES, SMP iPS or Sca1+ iPS cells.

**Figure 6:**
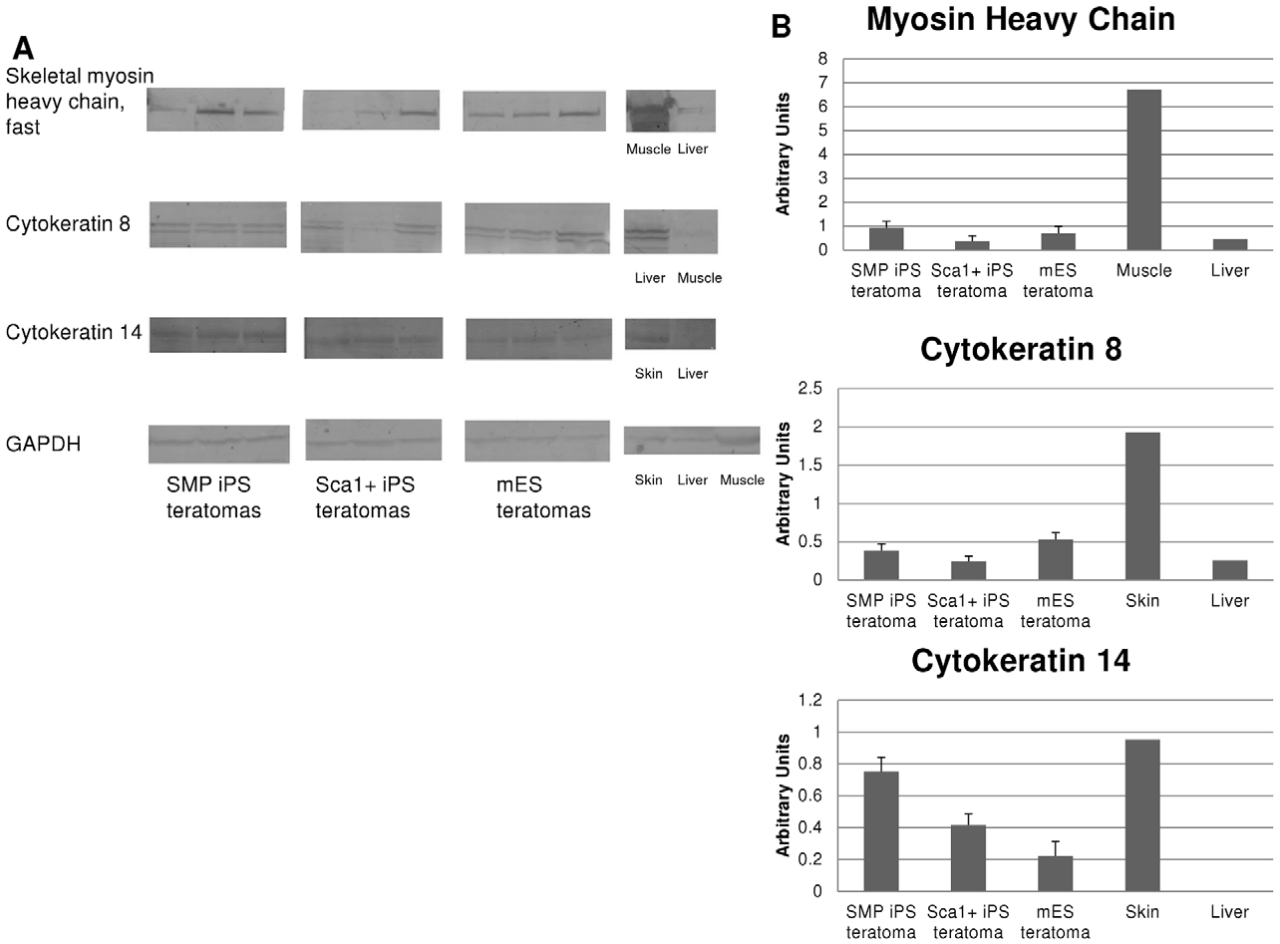
Teratomas from SMP iPS and Sca1+ iPS cells do not show biased differentiation into skeletal muscle by Western blot. (**A**) Representative Western blots of teratomas from SMP iPS, Sca1+ iPS, or mES cells, stained with the indicated antibodies against skeletal muscle myosin heavy chain (MyHC), cytokeratin 8 (CK8), cytokeratin 14 (CK14) or GAPDH as a control. Three different lines were used for each pluripotent cell type to generate the teratomas. (**B**) Western blots were quantified by optical densitometry using ImageJ, and results reported for MyHC (top), CK8 (middle) and CK14 (bottom) as mean intensity (arbitrary units), normalized to the loading control (GAPDH). Error bars reflect standard error. No significant differences were detected in the presence of MyHC (a mesodermal and skeletal muscle marker) or CK8 (an endodermal marker) among any of the three sources of pluripotent cells. SMP iPS showed 2–3 fold greater representation of CK14 (an ectodermal marker) than Sca1+ iPS or mES. N.D. – not detectable.

Teratomas were obtained from injection of each pluripotent cell line, and harvested when they reached a diameter of ∼0.5 cm, typically 14–21 days after injection. No differences in tumor size were observed for iPS- vs. ES-derived teratomas (data not shown). In addition, in all cases, the injected cells formed tumors containing cells from all three germ layers, a pattern of cell fate specification consistent with the pluripotent nature of iPS and mES cells (Figure 5A and 6). Histological assessment confirmed the presence of neural cells, cartilage, adipose, columnar epithelium and keratinocytes in all teratomas (Figure 5A and data not shown); however, in all cases, skeletal muscle comprised only a small fraction (2–4%) of tumor mass (Figure 5B). Importantly, teratomas derived from SMP iPS cells did not generate skeletal muscle to a significantly higher degree than teratomas initiated by Sca1+ cell-derived iPS, or by mES, despite the clearly superior myogenic differentiation potential of SMPs as compared to other cell types present in skeletal muscle ([2], [4], and Figure 1B). Moreover, although Sca1+ cells possess no intrinsic myogenic capacity when isolated from skeletal muscle (Figure 1B and [2], [3], [4], [6]), Sca1+ cell derived iPS showed no impairment in differentiation to the muscle lineage in these teratoma assays (Figure 5B). Overall, teratomas derived from Sca1+ iPS, SMP iPS, and mES cells contained an equivalent representation of muscle cells as assessed by histology (Figure 5B). This semiquantitative observation was corroborated by Western blot analysis, which showed no significant differences in the presence of skeletal muscle myosin heavy chain in teratomas derived from Sca1+ iPS, SMP iPS, or mES cells (Figure 6B), indicating equivalent differentiation along the skeletal muscle lineage. Furthermore, teratomas from Sca1+ iPS, SMP iPS, or mES cells each contained mesoderm (indicated by myosin heavy chain, MyHC), ectoderm (indicated by cytokeratin 14, CK14) and endoderm (indicated by cytokeratin 8, CK8) (Figure 6A) at roughly equivalent levels (Figure 6B). These data indicate that none of the pluripotent stem cell types generated here were deficient in differentiation into any of the three germ layers. These data argue that although early passage SMP iPS cells may retain increased expression of some mRNAs associated with their somatic cell origin (e.g., β1-integrin and CXCR4; [8]), iPS cell epigenetic memory is not in this case sufficient to drive differentiation of teratoma cells down a predominantly skeletal muscle fate.

## Discussion

Taken together, our observations that SMPs and Sca1^+^ mesenchymal progenitors reprogram to generate iPS cells with greater efficiency than differentiated CXCR4^−^ cells suggests that pluripotency can be induced more efficiently in stem cells as compared to their more differentiated progeny residing within the same anatomical location and same developmental lineage. In combination with a recent report [7], our data suggest that increased susceptibility to reprogramming may be a common attribute of many adult stem cells. These data thus emphasize the crucial impact of differentiation stage on reprogramming efficiency, and indicate that much higher efficiencies of iPS derivation can be obtained simply by selecting an appropriate target population; using undifferentiated precursor cells as targets for reprogramming substantially enhances the efficiency of iPS generation. Future studies identifying the molecular differences between immature and mature cell populations should reveal critical barriers inherent to the reprogramming process and thereby facilitate effective generation of iPS cells for disease modeling, drug discovery and cell therapy approaches.

**Figure 7:**
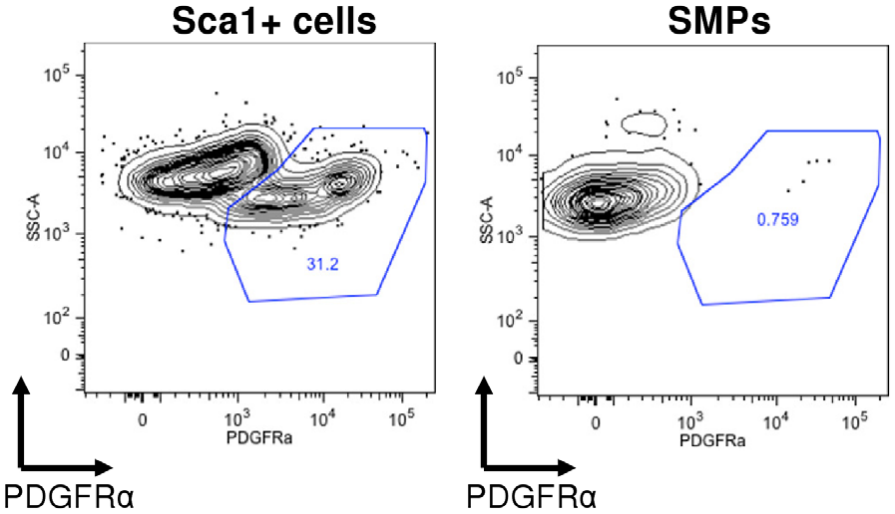
Sca1+ cells but not SMPs contain a PDGFRα+ population. Sca1+ cells or SMPs were stained for PDGFRα and analyzed by FACS. Representative FACS plots show that only Sca1+ cells (left) contain a substantial population of PDGFRα+ cells (∼30% of Sca1+ cells), whereas SMPs (right) are PDGFRα−.

Our data also indicate that, despite some epigenetic memory of their somatic cell of origin [8], SMP iPS show no myogenic bias in their differentiation in teratoma assays, as assessed by both histological analysis and Western blotting for lineage-specific differentiation markers. Similarly, although Sca1+ cells are uniformly non-myogenic in vivo and in vitro, iPS cells generated from Sca1+ cells differentiate to form skeletal muscle as efficiently as SMP iPS. Intriguingly, these findings contrast with a recent report that mesangioblast-derived iPS cells (MAB-iPS) generate teratomas containing up to 70% striated muscle [18]. Differences in these results may reflect differences in the degree of lineage specification among these two cells types, as mesangioblasts have been reported to retain both myogenic and non-myogenic differentiation capacity (including the ability to differentiate to form endothelium, fibroblasts, bone, fat, cartilage and neural cells), while SMPs represent lineage-committed muscle stem cells [2]. Alternatively, it is possible that the serial passaging and selection employed in the culture-based isolation of mesangioblasts introduces changes in these cells that influence the subsequent activity of iPS cells derived from them. Of note, the mesangioblasts (MAB) that Quattrocelli et al isolated were obtained from murine skeletal muscle and reported to derive from Sca1+ and PDGFRα+ cells. Whereas the freshly isolated SMPs studied here are uniformly Sca1− and PDGFRα−, the “Sca1+ cells” in our isolations contain ∼30% PDGFRα+ cells (Figure 7), suggesting that the Sca1+ population we studied is likely to contain a substantial fraction of mesangioblasts. Nonetheless, in our studies, Sca1+ iPS, like SMP iPS, generated teratomas containing only ∼5% skeletal muscle (by surface area), with no differences in the presence of skeletal muscle myosin heavy chain (determined by Western blotting and densitometry). Thus, unlike prior studies of iPS derived from mouse hematopoietic cells [8], and recent studies of iPS derived from human pancreatic islets [9], each of which reported an increased propensity of iPS to differentiate into somatic cells of the same lineage as the iPS cell-of-origin, our data indicate that the myogenic potential of the target cell is insufficient, in and of itself, to impose subsequent differentiation biases on early passage reprogrammed iPS cells. These findings are consistent with a model in which cell-of-origin is only one of many possible influences on the differentiation potential of iPS cells derived from distinct somatic cells, and may reflect an incompatibility of the myogenic program with pluripotency that may help to explain difficulties encountered in attempts to direct the differentiation of these cells along the skeletal muscle lineage. Thus, while differentiation biases imparted to iPS cells based on their cell-of-origin may be useful for deriving some somatic tissue types, they may not provide a universal advantage for directing pluripotent cell differentiation.

## Materials and Methods

### SMP isolation

Myofiber-associated cells were prepared from intact limb muscles (EDL, gastrocnemius, quadriceps, soleus, TA, and triceps brachii) as described previously [2], [19]. Briefly, intact mouse limb muscles were digested with collagenase II to dissociate individual myofibers. These were triturated and digested with collagenase II and dispase to release myofiber-associated cells. The myofiber-associated cells were next subfractionated by FACS, using the following marker profiles for each population: 1) SMPs: CD45^−^ Mac-1^−^ Sca-1^−^ β1-integrin^+^ CXCR4^+^; 2) Myoblast and fibroblast-containing population: CD45^−^ Mac-1^−^ Sca-1^−^CXCR4^−^; 3) Sca1^+^ mesenchymal cells: CD45^−^ Mac-1^−^ Sca-1^+^. After the initial sort, cells were re-sorted by FACS using the same gating profile to increase the purity of the obtained population [20].

### Reprogramming of myofiber-associated cells

Sorted cells from mice containing dox-inducible Oct4, Sox2, c-Myc and Klf4 transgenes were cultured on irradiated MEFs, in ES medium in the presence of doxycycline (10 ug/ml; Sigma D9891-25). Doxycycline was removed once emergent iPS colonies became stably reprogrammed (generally 21–28 days).

### Generation of reprogrammable mice

Generation of the reprogrammable mouse was described in [11], [12], [13]. Briefly, iPS cells were initially derived from a neonatal tail fibroblast-derived iPS clone that was injected into blastocysts to produce fetal liver cells. The iPS-derived fetal liver cells were harvested and differentiated in vitro into CD8+ cells, which then were re-induced with dox to produce iPS cells. These secondary iPS cells were then injected into blastocysts to generate reprogrammable mice as used in this study.

### Quantitative PCR

SMPs, Sca1+ and CXCR4− cells were harvested from mouse skeletal muscle, and double-sorted for purity. After the second sort, cells were deposited in Trizol and cDNA was prepared using Superscript III Reverse Transcriptase Supermix kit (Invitrogen, 11752-050). Real-time quantitative PCR reactions were carried out in an ABI 7900 machine, using SYBR Green PCR mix (Applied Biosystems, 4309155). Beta 2 microglobulin (B2M) was used as a housekeeping gene, and gene expression levels normalized to B2M expression. Primers used to amplify endogenous genes were (listed 5′ to 3′; sequences from [14]):

c-Myc: AAGAGGACTTGTTGCGGAAA and TTGTAATCCAGAGGTTGATTATCG

Klf4: ATGGTCAAGTTCCCAGCAAG and TGATATCGAATTCCGTTTGTTT

Oct4: AGTTGGCGTGGAGACTTTGC and CAGGGCTTTCATGTCCTGG

Sox2: GGCCATTAACGGCACACT and AAGCAGCGTATCCACATAGC

B2m: TTCTGGTGCTTGTCTCACTGA and CAGTATGTTCGGCTTCCCATTC

### Cell culture media

Embryonic stem cell media consisted of high glucose DMEM (Gibco 11965-092) supplemented with 15% ES-qualified fetal bovine serum (FBS; Gemini), 1 mM L-Glutamax (Invitrogen 35050-061), 100 U/ml penicillin and 100 U/ml streptomycin (Invitrogen 15070-063), 1 mM sodium pyruvate (Invitrogen 11360-070), 1× non-essential amino acids (Invitrogen 11140-050), 0.1 mM ß-mercaptoethanol (Invitrogen 21985-023), 1000 units/ml LIF (ESGRO, Millipore).

### Immunostaining and alkaline phosphatase staining

Immunostaining was performed with antibodies to Oct4 (Abcam ab19857) and SSEA-1 (Chemicon MAB4301). Alkaline phosphatase staining was performed using the Leukocyte Alkaline Phosphatase kit from Sigma (86R-1KT) according to manufacturer's instructions.

#### Myogenic differentiation assays

Freshly sorted cells were plated at 1×10^4^ cells/ well in 96 well plates, coated with 1 µg/ml rat-tail collagen (Sigma) and 10 µg/ml natural mouse laminin (Invitrogen). Cells were expanded for 5–7 days in growth medium (GM) composed of Ham's F10 (Invitrogen)+20% horse serum (Atlanta Biologicals)+1% penicillin/ streptomycin+5 ng/ml bFGF (Sigma). bFGF was replaced daily. After 5–7 days, cells were passaged and replated onto 0.2% Matrigel (Fisher) coated chamber slides in growth medium for 2 days, and then medium was changed to differentiation medium (DM) consisting of Ham's F10+2% horse serum+1% penicillin/ streptomycin. Cells were kept in DM for 6 days, then medium was aspirated and cells were fixed with 4% paraformaldehyde and processed for immunofluorescence [4].

#### Adipogenic differentiation assay

Freshly sorted cells were expanded as described above. After 5–7 days, cells were replated onto 0.2% Matrigel coated 48-well plates in growth medium for 2 days. Subsequently, medium was changed to adipogenic induction medium consisting of DMEM+10% FBS+1% penicillin/ streptomycin+1 mM Dexamethasone (Sigma)+100 nM Insulin (Sigma)+1 mM Roziglitazone (Cayman Chemical)+0.5 mM 3-isobutyl-1-methylxanthine (Sigma) for 2 days, and then replaced with adipogenic differentiation medium consisting of DMEM+10% FBS+1% penicillin/ streptomycin+100 nM Insulin. Cells were kept in this medium for 6 days, and then fixed with 4% Paraformaldehyde and stained with Oil Red O (Sigma) for one hour at room temperature [21]. Oil Red O staining of lipid droplets within adipocytes was analyzed by standard microscopy using an Olympus IX51 inverted microscope.

#### Teratoma formation

To generate teratomas, pluripotent cells were injected subcutaneously into the back of NOD/SCID mice. Mice were sacrificed when tumors reached approximately 0.5 cm in diameter, and tumors were harvested for fixation and sectioning, or for protein analysis via western blots.

#### Western blots

Teratomas were flash frozen in liquid nitrogen, and homogenized in buffer containing protease inhibitors (cOmplete protease inhibitors, Roche). The homogenate was centrifuged, and supernatant taken for protein analysis. Protein concentration was determined using a BCA protein assay kit (Pierce), and samples were run on a 4–20% polyacrylamide gel (Criterion, BioRad). Proteins were transferred onto a PVDF membrane (Immuno-Blot, BioRad), and blocked with 5% nonfat dry milk. Primary antibody staining was done in Tris-buffered saline (Atlanta Biologicals) with 0.1% Tween-20 containing 5% milk at 4°C overnight on a rocking platform. Secondary antibody staining was done in 5% milk at room temperature for 1 hour on a rocking platform. Protein bands were visualized using enhanced luminescence reagents (Amersham RPN2232) and quantified using a Typhoon Imager (Amersham). Band intensities were quantified using ImageJ (National Institutes of Health). Antibodies used were as follows: Anti-GAPDH (Abcam ab9485), Anti-skeletal fast myosin heavy chain (Abcam ab91506), Anti-cytokeratin 14 (Abcam ab7800), Anti-cytokeratin 8 (Developmental Studies Hybridoma Bank, Troma-1), Anti-Mouse IgG Peroxidase Conjugated (Thermo Scientific 31430), Anti-Rabbit IgG Peroxidase Conjugated (Thermo Scientific 31460), Anti-Rat IgG Peroxidase Conjugated (Thermo Scientific 31470). Optical densitometry was done using ImageJ according to http://lukemiller.org/index.php/2010/11/analyzing-gels-and-western-blots-with-image-j/.

### Animal welfare

This study was carried out in strict accordance with the recommendations in the Guide for the Care and Use of Laboratory Animals of the National Institutes of Health. The protocol was approved by the Massachusetts General Hospital Subcommittee on Research Animal Care (Protocol number 2006N000104 to Konrad Hochedlinger) and the Joslin Diabetes Center Institutional Animal Care and Use Committee (Protocol 04-01 to Amy Wagers) and Harvard University Standing Committee on the Use of Animals in Research and Teaching (Protocol 29-14 to Amy Wagers). All surgeries were performed under anesthesia, and all efforts were made to minimize suffering. Animals were humanely sacrificed prior to tissue collection. iPS cells were generated in accordance with the approved protocols from Massachusetts General Hospital (Protocol number 2006N000104 ), Joslin Diabetes Center (Protocol 04-01) and Harvard University (Protocol 29-14) as stated above.

## Supporting Information

Figure S1 **Generation of “reprogrammable mice” and experimental design to test reprogramming efficiency of myofiber-associated cells.** Induced pluripotent stem cells (iPS cells) were generated from mouse tail-tip fibroblasts by infection with lentiviruses containing doxycycline-inducible Oct4, Sox2, Klf4 and c-myc transgenes. Addition of doxycycline (dox) induces reprogramming. These iPS cells were injected into e3.5 mouse blastocysts, where they contributed to fetal liver. Fetal liver cells were harvested, differentiated into CD8+ cells, and dedifferentiated again using dox. The resulting iPS cells were used to generate the reprogrammable mice for this study. The limb muscles of these reprogrammable mice were harvested to myofiber-associated cells, which were cultured in the presence of dox to produce tertiary iPS cells. Muscle-derived iPS cells were used to generate transgenic mice, which when bred to wild-type mice demonstrated germline transmission.
(PDF)

Figure S2 **Bulk-sorted SMPs and Sca1+ cells reprogram more efficiently compared to CXCR4− myoblasts.** (**A**) Myofiber-associated cells were isolated from chimeric mice, transgenic for dox-inducible Oct4, Sox2, Klf4 and c-myc (but not tdTomato). Doxycycline was added to induce transgene expression of Oct4, Sox2, Klf4 and c-myc. These cells consist of a mix of transgenic and non-transgenic cells; therefore, analysis of these cell populations, underestimates the reprogramming efficiency of each cell type. Reprogramming efficiencies reported in (**B**) are given as the percent of input cells (per number of cells seeded) generating colonies with embryonic stem cell-like morphology after 3 weeks. ND: not determined.
(PDF)

Figure S3 **Representative FACS plots of tdTomato+ cells sorted from the myofiber-associated cell compartment for reprogramming.** FACS gating of the indicated cell populations is indicated by blue boxes and red arrows. The percent of cells within each gate is as shown. tdTomato-expression indicates the presence of cells transgenic for the four dox-inducible reprogramming factors in all the populations (Sca-1+ (top), CXCR4− (middle), and SMP (bottom)).
(PDF)
