# Supplementary figures and images for: Efficient Generation of iPS Cells from Skeletal Muscle Stem Cells
Source: PLoS One. 2011 Oct 18;6(10):e26406. doi: 10.1371/journal.pone.0026406 (PMC3196574; doi:10.1371/journal.pone.0026406)

FIGURE S1

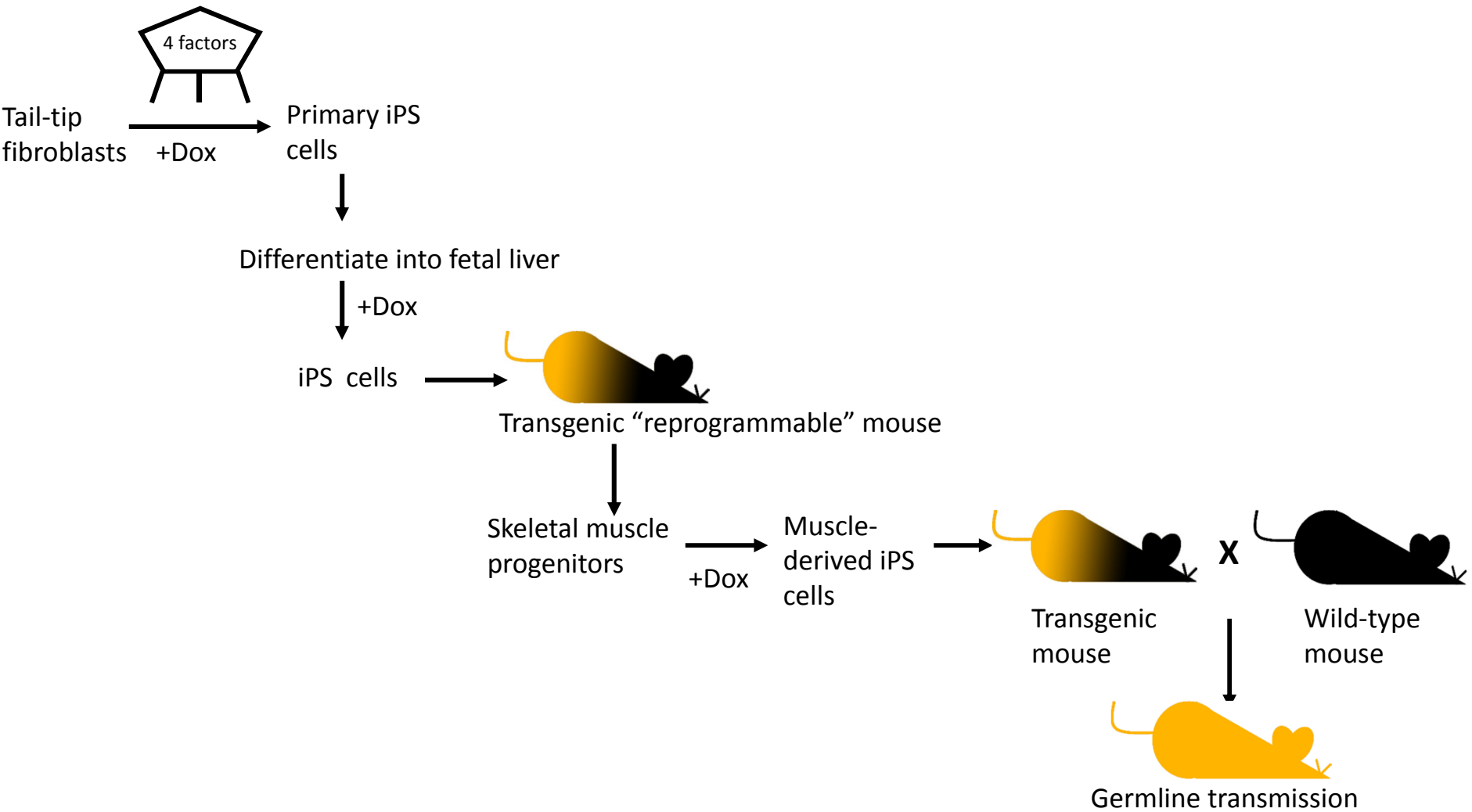

Supplement: Figure S1 — Generation of “reprogrammable mice” and experimental design to test reprogramming efficiency of myofiber-associated cells. Induced pluripotent stem cells (iPS cells) were generated from mouse tail-tip fibroblasts by infection with lentiviruses containing doxycycline-inducible Oct4, Sox2, Klf4 and c-myc transgenes. Addition of doxycycline (dox) induces reprogramming. These iPS cells were injected into e3.5 mouse blastocysts, where they contributed to fetal liver. Fetal liver cells were harvested, differentiated into CD8+ cells, and dedifferentiated again using dox. The resulting iPS cells were used to generate the reprogrammable mice for this study. The limb muscles of these reprogrammable mice were harvested to myofiber-associated cells, which were cultured in the presence of dox to produce tertiary iPS cells. Muscle-derived iPS cells were used to generate transgenic mice, which when bred to wild-type mice demonstrated germline transmission. (PDF) [file pone.0026406.s001.pdf]

FIGURE S3

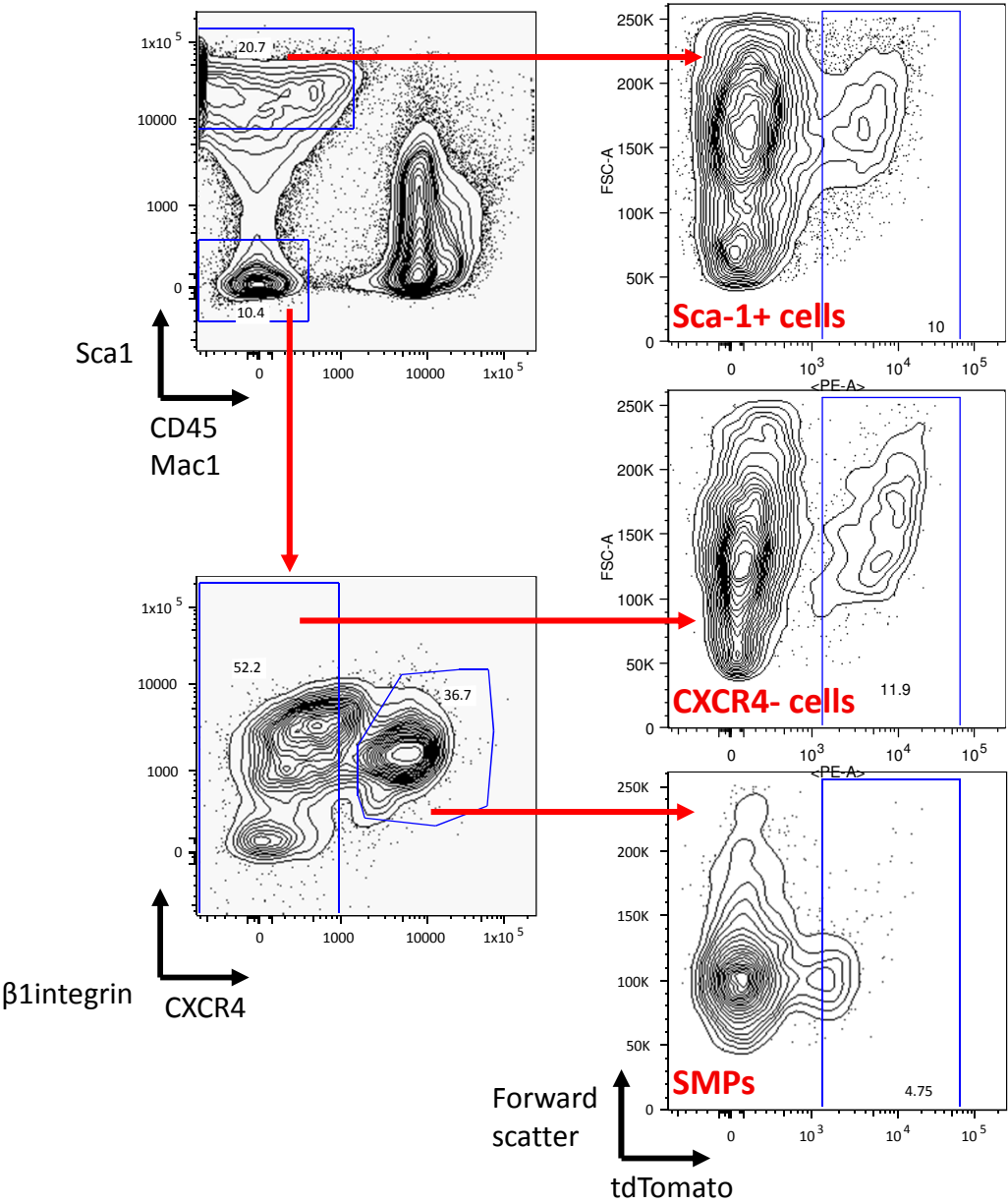

Supplement: Figure S3 — Representative FACS plots of tdTomato+ cells sorted from the myofiber-associated cell compartment for reprogramming. FACS gating of the indicated cell populations is indicated by blue boxes and red arrows. The percent of cells within each gate is as shown. tdTomato-expression indicates the presence of cells transgenic for the four dox-inducible reprogramming factors in all the populations (Sca-1+ (top), CXCR4− (middle), and SMP (bottom)). (PDF) [file pone.0026406.s003.pdf]
