# Supplementary material for: Efficient Generation of iPS Cells from Skeletal Muscle Stem Cells
Source: PLoS One. 2011 Oct 18;6(10):e26406. doi: 10.1371/journal.pone.0026406 (PMC3196574; doi:10.1371/journal.pone.0026406)
Supplement: Figure S2 — Bulk-sorted SMPs and Sca1+ cells reprogram more efficiently compared to CXCR4− myoblasts. (A) Myofiber-associated cells were isolated from chimeric mice, transgenic for dox-inducible Oct4, Sox2, Klf4 and c-myc (but not tdTomato). Doxycycline was added to induce transgene expression of Oct4, Sox2, Klf4 and c-myc. These cells consist of a mix of transgenic and non-transgenic cells; therefore, analysis of these cell populations, underestimates the reprogramming efficiency of each cell type. Reprogramming efficiencies reported in (B) are given as the percent of input cells (per number of cells seeded) generating colonies with embryonic stem cell-like morphology after 3 weeks. ND: not determined. (PDF) [file pone.0026406.s002.pdf]

FIGURE S2

A

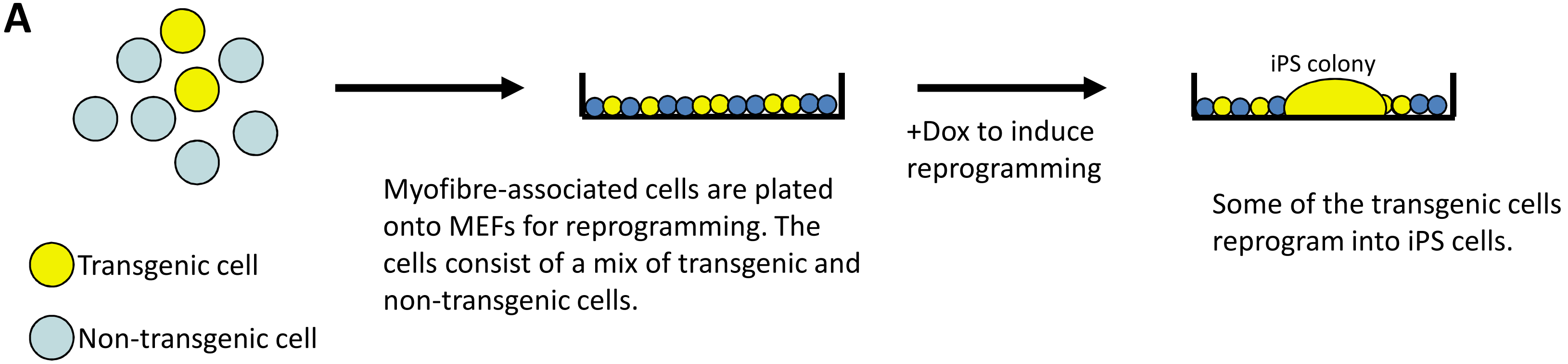

B

Cell type	Efficiency of reprogramming X% (No of colonies / No of cells seeded)	
	Expt 1	Expt 2
Skeletal muscle progenitors (Sca1- CD45- Mac1- B1-integrin CXCR4-; "SMPs" in text)	0.7% (15/2100)	2.8% (38/1352)
Mesenchymal progenitors (Sca1+ CD45- Mac1-; "Sca1+ cells" in text)	0.01% (2/2100)	0.07% (1/1352)
Myoblast-containing population (Sca1- CD45- Mac1- CXCR4-; "CXCR4- cells" in text)	ND	0% (0/1352)
